# External beam radiotherapy inhibits stent related granulation hyperplasia in rabbit trachea

**DOI:** 10.1038/s41598-023-34449-5

**Published:** 2023-05-03

**Authors:** Zihe Zhou, Bin Han, Kewei Ren, Yahua Li, Kunpeng Wu, Janan Wang, Yifan Li, Zongming Li, Xinwei Han

**Affiliations:** 1 Department of Interventional Radiology, The First Affiliated Hospital of Zhengzhou University, Zhengzhou, 450052 Henan People’s Republic of China; 2 Department of Radiotherapy, The First Affiliated Hospital of Zhengzhou University, Zhengzhou, 450052 People’s Republic of China; 3 Interventional Treatment and Clinical Research Center of Henan Province, Zhengzhou, 450052 Henan People’s Republic of China; 4 Interventional Institute of Zhengzhou University, Zhengzhou, 450052 Henan People’s Republic of China

**Keywords:** Preclinical research, Experimental models of disease

## Abstract

Endobronchial stent exacerbates the formation of granulation tissue. Radiotherapy maybe a durable treatment option for granulation hyperplasia. In this study, we explore the results of external beam radiotherapy (EBRT) for granulation hyperplasia after airway stent placement. A total of 30 New Zealand rabbits were assigned in three groups, Control group (n = 12), low dosage (LD, 12 Gy in 4 fractions and twice a week) group (n = 9) and high dosage (HD, 20 Gy in 4 fractions and twice a week) group (n = 9). Post-stenting 1 week, LD and HD group started to receive EBRT. Bronchoscopy, Haematoxylin–eosin (HE), Masson’s trichrome (MTS), Safranin O (SO) and immunohistochemical (IHC) staining protocols were performed to evaluate the histopathological changes of trachea. A total of 30 stents were successfully implanted in 30 rabbits. No procedure-related death and complications happened. Post-stenting 4 w, 8 w and 12 w, the ventilate area ratio (VAR) and qualitative histological scoring (QHS) in the LD group and HD group lower than the Control group. Post-stenting 12w, the immunohistochemical results revealed that the positive percentage of TGF-β and VEGF in the LD group and HD group were lower than the Control group. In conclusion, the present study investigated the efficacy of EBRT in reducing stent related granulation tissue formation in the rabbit trachea. Higher dosage EBRT with a better result in inhibiting granulation hyperplasia.

## Introduction

Airway stenting is an effective way to restore patency. The placement of stents significantly improved outcomes of patient with airway stenosis. However, stents themselves are not without complications and do not represent a definitive treatment.

Potential complications related to non-removability stent mainly exacerbate the formation of endobronchial granulation tissue. Long-term effectiveness and sustainability are hampered by tissue responses after stenting^1–3^.

Endobronchial granulation tissue hyperplasia around the stent is formed as partial natural wound healing process. The main components of granulation tissue are accumulated inflammatory cells, newly formed connective tissue and small blood vessels^4^. It often presents asymptomatically, however, proliferation of endobronchial granulation tissue may cause a decline in oxygen saturation even worsening pulmonary function tests. Which frequently associated with loss of treatment effectiveness^1,3,5^. Furthermore, it is one of the most common reasons for additional bronchoscopy procedures, which form a risk and burden for the patient.

Radiotherapy has widely used in malignant tumor treatment, also in benign keloids. Mandu et al.^4^ reported the experience of 5 patients with significant airway compromise from recurrent granulation tissue treated with high-dose-rate ^192^Ir brachytherapy. The total doses ranging from 10 to 21 Gy in two to three fractions. During the 12 months follow-up, 3 of the 5 patients alive with marked symptomatic improvement and reduced bronchoscopy procedures. These benign conditions exhibit a good and durable response to radiation, which has the ability to inhibit growth of benign tissue proliferation^4,6^. As a subtype of benign keloids, the reactive granulation tissue hyperplasia in the airway may have a similar reaction to radiation. Therefore, radiotherapy maybe a durable treatment option for the treatment of granulation hyperplasia after stent placement. In this study, we explore the results of external beam radiotherapy (EBRT) for granulation hyperplasia after airway stent placement.

## Results

A total of 30 stents were successfully implanted in 30 rabbits. The stents were released by a 6 F delivery system under the guidance of fluoroscopy. No procedure-related complications happened. The rabbit in the LD group and HD group successfully received EBRT. No radiotherapy related-death happened.

### Bronchoscopy

**Figure 1:**
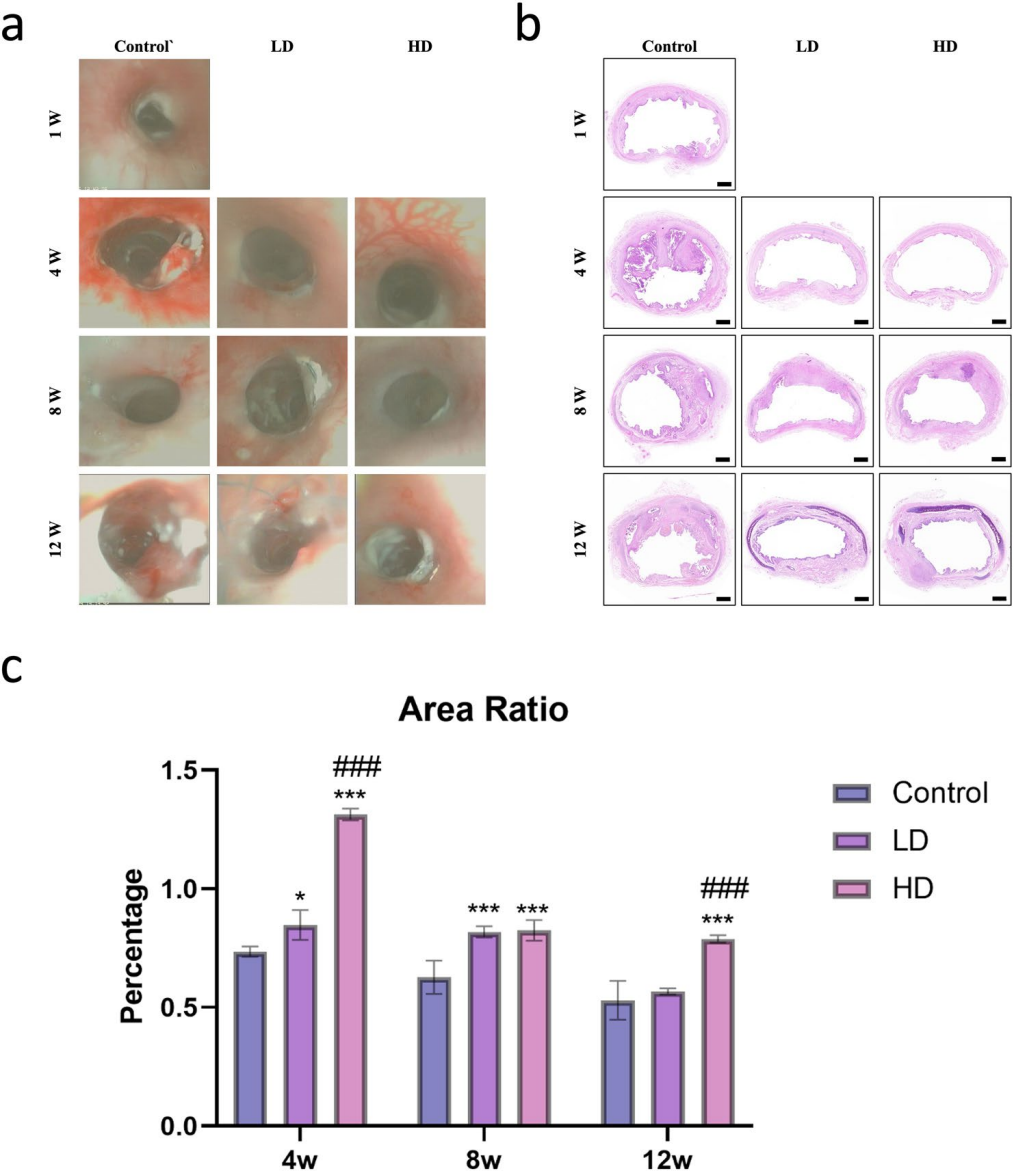
The represent images of bronchoscopy (**a**) and HE staining (**b**) on 1w, 4w, 8w and 12w follow up. The VAR was calculated and analysed. (**c**) Bar = 1 mm. “*” stand for compared to Control group. “#” stand for compared to LD group. P-values < 0.05 (*), < 0.01 (**), and < 0.001 (***). P-values < 0.001 (###).

Representative bronchoscopic images of post-stenting are shown in Fig. 1a. Post-stenting 1w, the proximal part of stented trachea was observed mildly reactive tissue response and sputum retention. Post-stenting 4 w, papillary projections were formation around the edge of proximal part of the stent in the Control group. After clearance the sputum, mild bleeding was observed from the papillary projections. Mildly reactive tissue response and sputum retention was observed in LD and HD group. Post-stenting 8 w, the edge of the proximal stent was embedded in the Control group, mild granulation hyperplasia and sputum retention was observed in LD and HD group. Post-stenting 12 w, the stent was severe embedded in the Control group. Small polyps were observed on the edge of the stent in LD and HD group.

### Ventilate area ratio

The stented trachea cross-section with HE staining images show in Fig. 1b. Post-stenting 4w, the VAR in Control group, LD group, and HD group are 0.74 ± 0.02, 0.85 ± 0.05, and 1.31 ± 0.02. Compared to Control group, there is significant difference in LD (P = 0.02) group and HD group (P < 0.001). There was statistical difference between the LD group and HD group (P < 0.001). Post-stenting 8w, the VAR in Control group, LD group, and HD group are 0.63 ± 0.06, 0.82 ± 0.02, and 0.82 ± 0.04. Compared to Control group, there is significant difference in LD (P < 0.001) group and HD group (P < 0.001). There was no statistical difference between the LD group and HD group (P = 0.984). Post-stenting 12w, the VAR in Control group, LD group, and HD group are 0.53 ± 0.07, 0.57 ± 0.01, and 0.79 ± 0.01. There was no statistical difference between the Control group and the LD group (P = 0.592). Compared to HD group, there is significant difference in the Control group (P < 0.001) and the LD group (P < 0.001) (Fig. 1c).

### Pathological analysis

**Figure 2:**
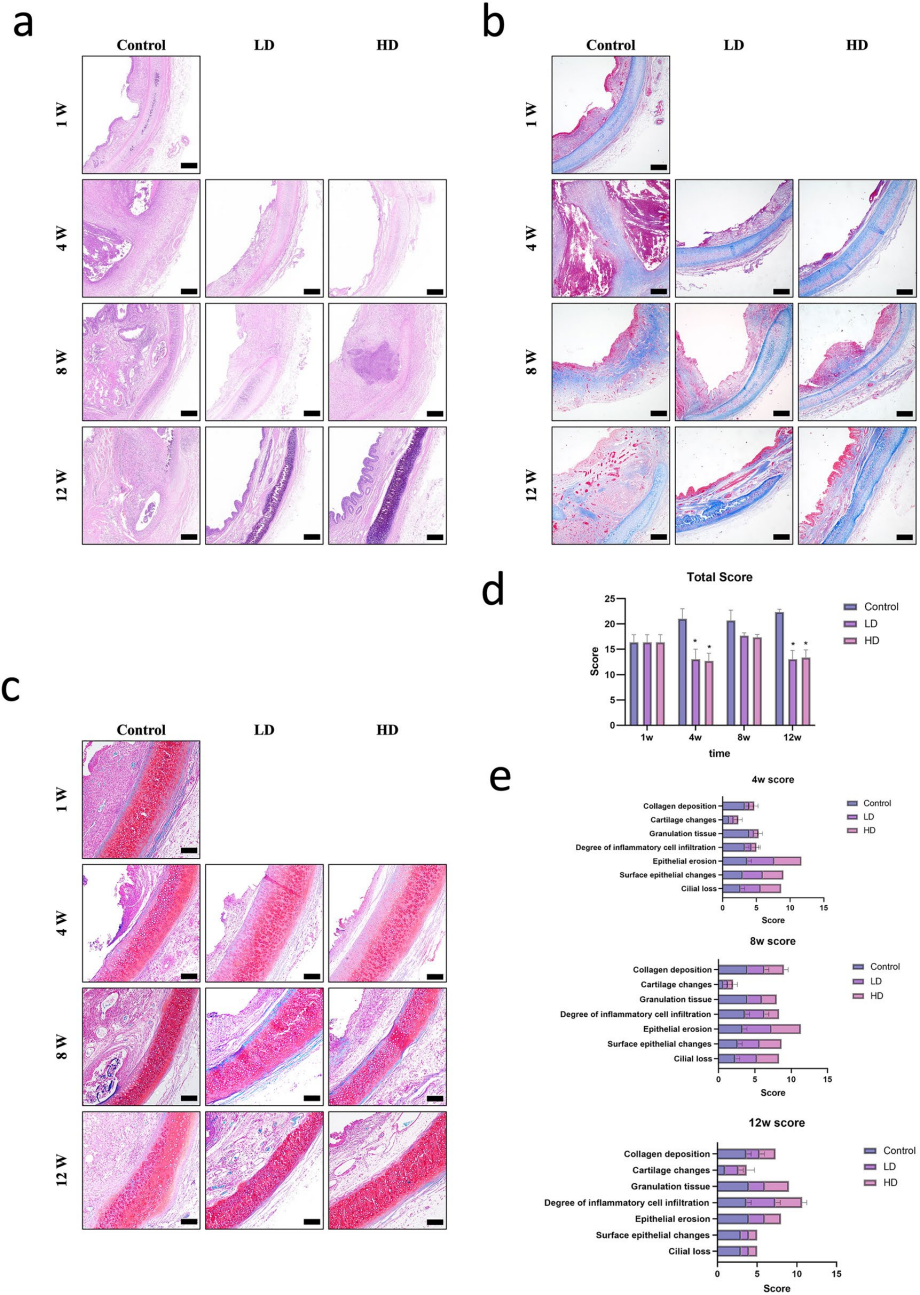
The represent images of HE (**a**), MTS (**b**), and OS (**c**) for details analysis. The total QHS analysis (**d**) and details analysis (**e**) in 3 groups on different follow-up time point. Bar = 200 μm. P-values < 0.05 (*), < 0.01 (**), and < 0.001 (***).

The represent images of HE, MTS, and OS for details analysis are shown in Fig. 2. Post-stenting 1w, the QHS in Control group is 16.33 ± 1.25. Post-stenting 4w, the QHS in Control group, LD group, and HD group are 21.00 ± 1.63, 13.00 ± 1.63, 12.67 ± 1.25. Compared to Control group, there is significant difference in LD (P = 0.037) group and HD group (P = 0.037). There was no statistical difference between the LD group and HD group (P = 0.645). Post-stenting 8w, the QHS in Control group, LD group, and HD group are 20.67 ± 1.70, 17.67 ± 0.47, 17.33 ± 0.47. There was no statistical difference among the 3 groups (Control vs LD; P = 0.169; Control vs HD; P = 0.226; LD vs HD; P = 0.645). Post-stenting 12w, the QHS in Control group, LD group, and HD group are 22.33 ± 0.47, 13.00 ± 1.41, 13.33 ± 1.25. Compared to Control group, there is significant difference in LD (P = 0.002) group and HD group (p = 0.003). There was no statistical difference between the LD group and HD group (P = 0.959) (Fig. 2d,e). The cartilage structure was observed by Safranin O staining, which did not show acidic proteoglycan staining in any of the groups. Every group could observe focal cartilage compression by stent.

**Figure 3:**
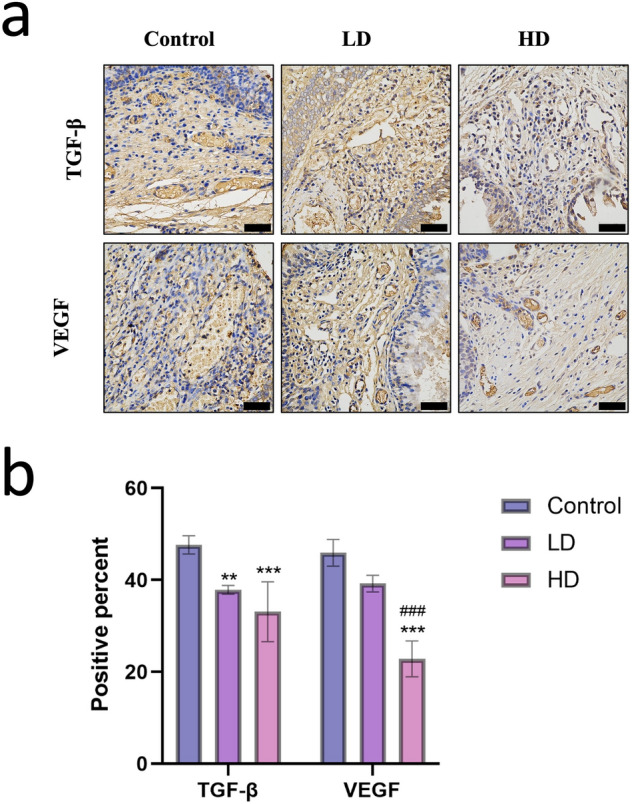
The immunohistochemical staining of TGF-β and VEGF on post-stenting 12w in 3 groups (**a**). The positive percentage analysis (**b**). Bar = 50 μm. “*” stand for compared to Control group. “#” stand for compared to LD group. P-values < 0.05 (*), < 0.01 (**), and < 0.001 (***). P-values < 0.001 (###).

Post-stenting 12w, the immunohistochemical results revealed the positive percentage of TGF-β in Control group, LD group, and HD group are 47.63 ± 1.61, 37.87 ± 0.78, and 33.10 ± 5.33. Compared to Control group, there is significant difference in LD (P = 0.007) group and HD group (P < 0.001). There was no statistical difference between the LD group and HD group (P = 0.187). The positive percentage of VEGF in Control group, LD group, and HD group are 45.93 ± 2.38, 39.23 ± 1.48, and 22.83 ± 3.18. There was no statistical difference between the LD group and Control group (P = 0.052). Compared to HD group, there is significant difference in the Control group (P < 0.001) and the LD group (p = 0.003) (Fig. 3).

## Discussion

Airway stenting is an effectively way to restore stenosis lumen, whether in benign or malignant airway stenosis. Stent related granulation tissue hyperplasia is a well-known complication that hampered their long-term effectiveness and sustainability^7^. In the treatment of malignant tracheal stenosis, stent placement combined with radiotherapy offered a long-term overall survival when compare stent placement only^6^. Inspired by these results, we designed this animal study to explore the results of different dosages radiotherapy for stent related granulation tissue hyperplasia.

In this study, we found that EBRT effectively reduce the growth of granulation. The ventilate area is the main evaluation parameter of stent-related granulation tissue hyperplasia. Post-stenting 1w, stent placement induced trachea tissue reaction boosts the formation of granulation tissue. Then, EBRT was performed in the LD group and HD group. Post-stenting 4w, both the LD group and HD group finished 4 times EBRT and rest 1 week. The VAR in HD group and LD group significantly higher than Control group. Which demonstrated the granulation tissue inhibit the effect of radiotherapy. However, in the HD group, the value of VAR larger than 1, which means that HD radiotherapy could reverse the granulation tissue hyperplasia. The QHS analysis suggested that collagen deposition, granulation tissue, and degree of inflammatory cell infiltration is the main 3 parameters affected by radiotherapy. Post-stenting 8w, the VAR in the HD group and LD group significantly higher than Control group. Although the QHS in the LD group and HD group lower than the Control group, but without statistical differences. The QHS analysis suggested that collagen deposition, granulation tissue, and degree of inflammatory cell infiltration is the main 3 parameters affected by radiotherapy. Post-stenting 12w, the VAR in HD group significantly higher than Control group and LD group. The QHS in the LD group and HD group lower than the Control group, with statistical differences. The QHS analysis suggested that collagen deposition, surface epithelial changes, and cilial loss is the main 3 parameters which related to the healing process.

Post-stenting healing process is a complicated condition that affects by inflammation, immune response, and mechanical stress. The injury of musca and cartilage are the key factors of benign airway stenosis formation^8^. There are three overlapping wound healing process, inflammation, proliferation, and remodelling^9^. The inflammation phase starts to post-stenting 1 day and sustained for 7 days. The proliferation phase of wound starts to post-stenting 4 days and sustained to 4 weeks. While the remodelling phase of wound-healing can last from months to years^10^. The application of radiotherapy effectively disturbed the process of post-stent healing process, especially the process of inflammation and proliferation. Which exhibit an anti-inflammatory effect. The mechanism of radiotherapy mitigates inflammation is via the polarization of macrophages to an anti-inflammatory or M2 phenotype^11^. Furthermore, the effect of radiation on tissues can be direct or indirect. The direct effect ionises DNA. It is the indirect effect through hydrolysis that is more harmful^12^. These characteristics explained the pathological response on post-stenting 4 weeks. Fibroblasts and neovascular are the main two components of granulation^13^. Fibroblasts bring the deposition collagen. The inhibit growth of fibroblasts caused the reduction of granulation volume and collagen deposition. Although the application of radiotherapy only 4 times and finished within 2 weeks, its long-term effects sustained not only within 12 weeks. In a clinical research, 43 patients received airway stenting followed by EBRT. EBRT had to be stopped prematurely in 37% patients, but the survival was poor, with a median overall survival (OS) of only 21 days. Twenty-seven patients completed radiotherapy as planned, with a median OS of 8.4 months^6^. Mallow and colleagues^14^ reported that patients who underwent stenting had an increased hazard ratio (HR) of death in comparison to those who received stenting combined EBRT. In this study, the HD group with a higher VAR when compare to Control group and LD group on post-stenting 12w. Which suggested that HD radiotherapy may have a longer long-term effect.

The profibrotic actions of transforming growth factor beta (TGF-β) have long made it a target of interest in the prevention of fibrosis, even in stent-related granulation hyperplasia^1,15,16^. Recent advances highlight that TGF-β is the master switch in pathogenesis of radiation fibrosis^9^. In this study, further IHC analysis suggested that EBRT significantly inhibit the expression of TGF-β in Control group. VEGF, another biomarker representing a neovascularization component, was also evaluated. Which has the similar result of TGF-β.

The safety and efficiency of this study also explored. No radiotherapy related death happened. The spinal cord is particularly sensitive to radiation therapy. Once the spinal cord is exposed to excessive radiation, the rabbit is prone to acute radioactive reactions leading to death. Therefore, when irradiating the trachea, the relative position of the spinal cord and trachea must be taken into account, and the dose of the spinal cord must be kept as low as possible when irradiating the trachea early, and the precision radiotherapy technology can achieve this goal well. As can be seen from the DVH diagram of the radiotherapy plan and the dose distribution diagram of the tracheal target area, the spinal cord radiation dose was significantly decreased, thus ensuring the safety of the experiment. Moreover, the high atomic number of the metallic component of stents can theoretically cause alterations in radiation dose from attenuation or backscatter effects. In the study of Evans and colleagues^17^, dosimeters were implanted in the tissues near and away from the stent. No significant difference was found in the dose perturbation factor for near and away dosimeters. External beam radiation can be delivered without concern for significant dose perturbation.

There are several limitations in this study. First, the small sample size of this study may affect the significance of the findings. Second, this study only explored two dosages of radiotherapy. Whether higher dosage radiotherapy bring a better result still require further explore. Finally, all rabbits in this study are health without airway stenosis. The establishment of airway stenosis model may contribute to further explore.

## Conclusion

In conclusion, the present study investigated the efficacy of EBRT in reducing stent related granulation tissue formation in the rabbit trachea. Higher dosage EBRT with a better result in inhibiting granulation hyperplasia.

## Methods

**Figure 4:**
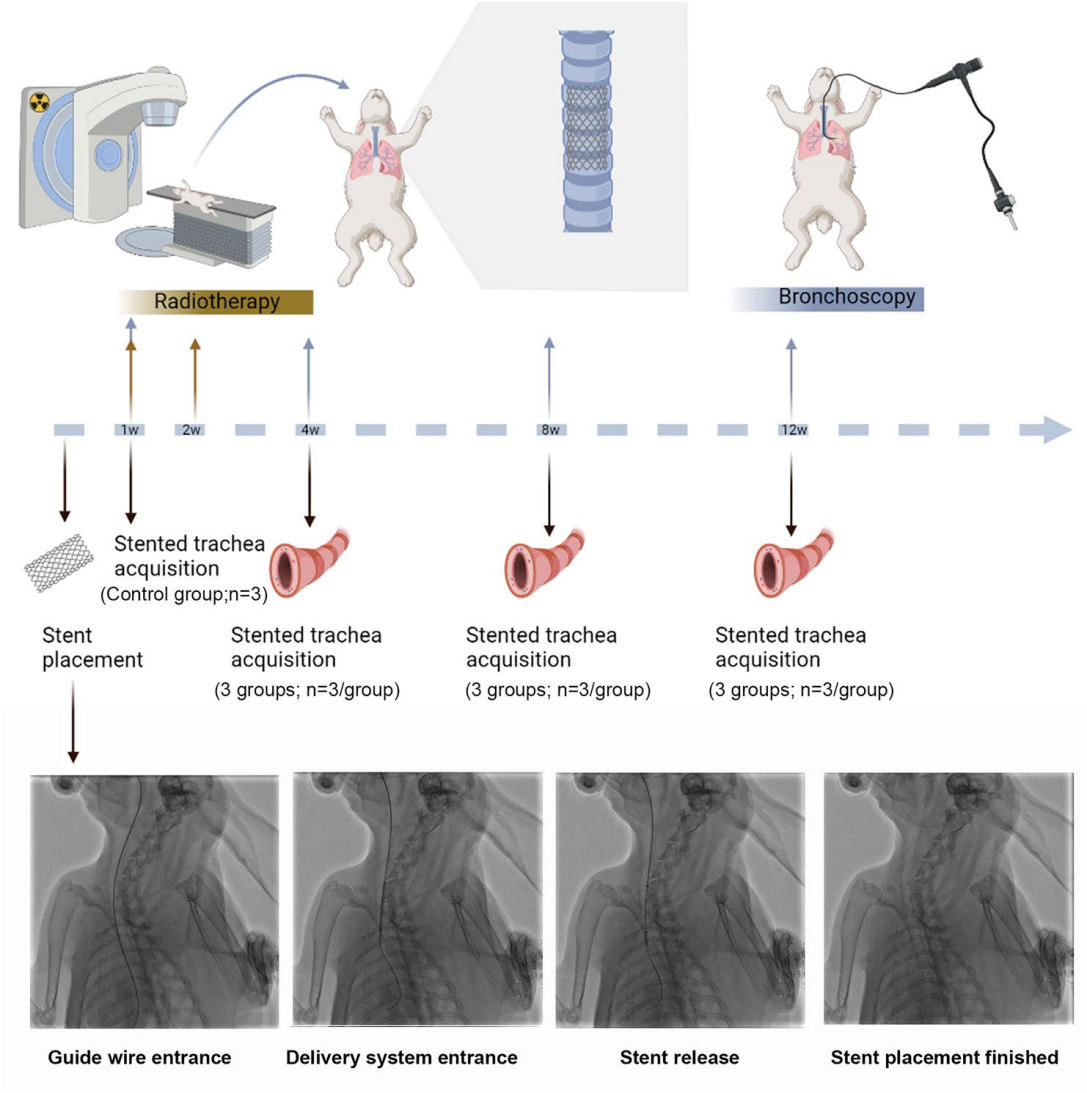
The plan, operation diagram, and procedure flow chart.

This animal study was reported in accordance with ARRIVE guidelines. A total of 30 New Zealand rabbits (both male and female) (3.0 ± 0.2 kg) were assigned in three groups, including Control group (n = 12), low dosage (LD) group (n = 9) and high dosage (HD) group (n = 9). The plan and operation flow chart are shown in Fig. 4. Xylazine Hydrochloride 1 mg/kg combined with pentobarbital sodium 0.5 mg/kg intramuscular injection was used for anesthesia. The stents were placed in the trachea under the guidance of fluoroscopy through a 6F delivery system (Fig. 4). All rabbits were maintained in single cages at a room temperature of 22 ± 2 °C, a relative humidity of 45 ± 15% and a 12-h light/dark cycle. Standard food and water could be accessed freely.

**Figure 5:**
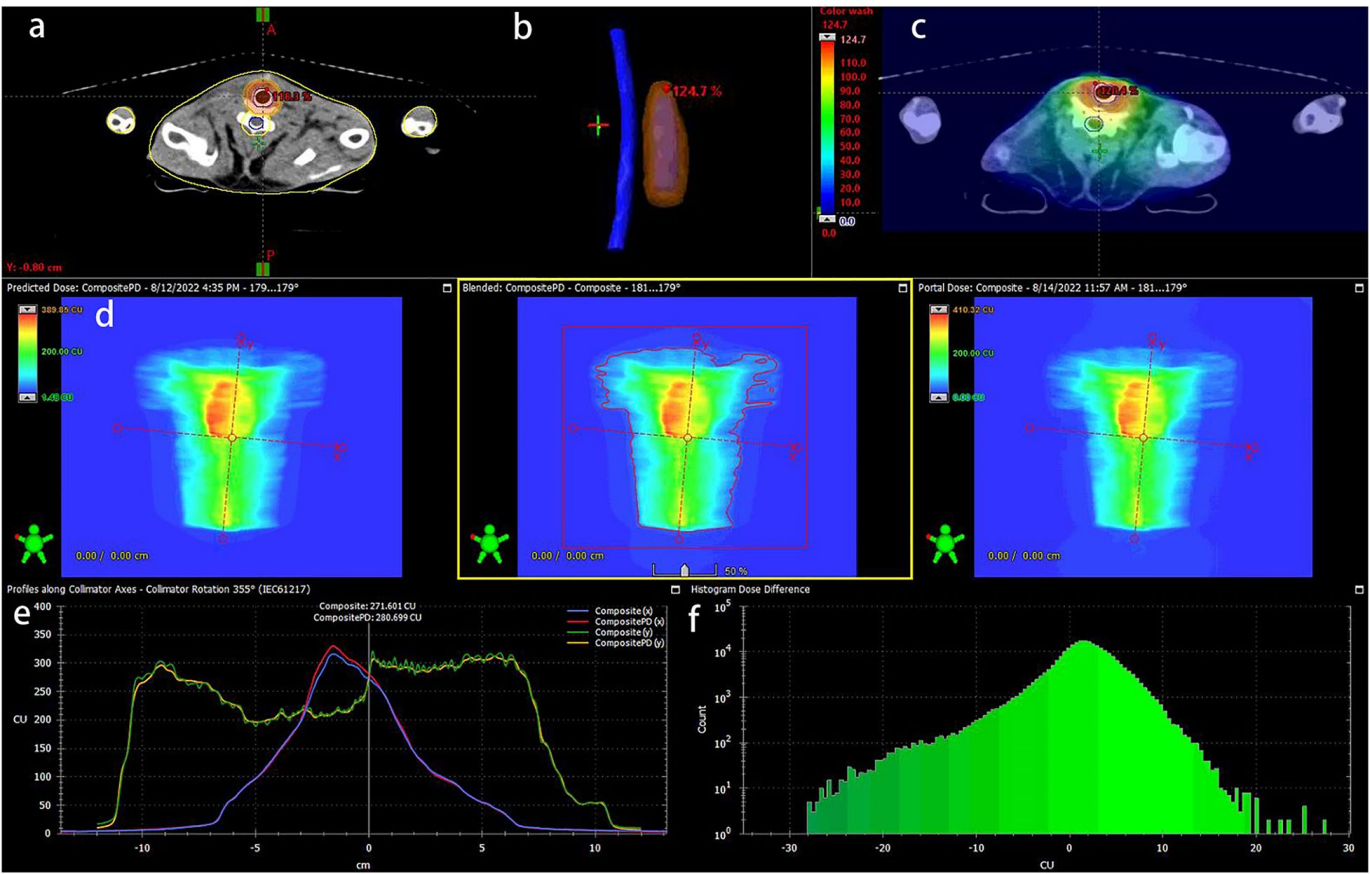
The process of EBRT. (**a**) Dose distribution in stented trachea. (**b**) The relative position of trachea and spinal cord. (**c**,**d**) The dose distribution diagram. (**d**) Planned dose—volume histogram of radiation therapy. (**e**,**f**) The dose-validation image of a radiation therapy plan.

Post-stenting 1 week, LD and HD group started to receive EBRT. After anesthesia, the rabbits were fixed in a plate. The CT simulator was utilized to perform radiotherapy localization, and delineated the area of airway stent implantation. The therapy planning system (TPS) was employed to design radiotherapy planning. Only the part of the trachea with stent was exposed to the specified dose. The dose of the surrounding tissues at risk was controlled, especially the dose of the spinal cord must be kept as low as possible. After the completion of the plan, dose verification was carried out to ensure that the pass rate was more than 90%, and then location verification was carried out. After all verification was passed, EBRT was performed for these rabbits (Fig. 5). The LD group treated with 12 Gy in 4 fractions and twice a week. The HD group treated with 20 Gy in 4 fractions and twice a week.

Bronchoscopy was performed to evaluate stent expansion and granulation hyperplasia at the post-stenting time points of 1 w, 4 w, 8 w and 12 w. The rabbits were sacrificed as schedule. Post-stenting 1w, 3 rabbits were sacrificed in Control group. The trachea with stents were sectioned transversely and fixed in 10% neutral buffered formalin for 24 h. Paraffin blocks with trachea were cut in 5-μm-thick sections to obtain slides. Haematoxylin–eosin (HE) staining was performed to analysis the changes of histopathology. Masson’s trichrome staining (MTS) was performed to analysis the collagen deposition. To evaluation the changes of tracheal cricoid cartilage, Safranin O (SO) staining protocols were performed. As key factors of granulation hyperplasia, the expression of TGF-β and VEGF were analysed by means of immunohistochemical staining. The slide and tissues were experienced pre-digestion, primary antibody incubation and second antibody incubation. The antibody of TGF-β (Anti-TGF beta 1 antibody; bs-0086R) and VEGF (Anti-VEGFA antibody; bs-1313R) were purchased from Bioss Biotechnology Co. LTD. Beijing China. Qualitative histological scoring (QHS) was performed using a stented tissue scoring system described in previous study^10,18^. The ventilate area ratio (VAR) was defined the value of ventilating area on each observation point by post-stent 1w. Analysis of the pathological results was based on the consensus of three observers who were blinded to the study.

### Statistical analysis

Differences between groups were analysed by analysis of variance, as appropriate. Post hoc comparisons were performed using the Bonferroni method. Fisher’s exact test and the chi-squared test were utilized to compare categorical variables between groups. Figures were made by GraphPad Prism 7.00 software. Statistical analysis was performed using SPSS 21.0, and P < 0.05 was considered statistically significant.

### Ethics approval and consent to participate

This animal study was approved by the Committee on the Ethics of Animal Experiments of Zhengzhou University-approval: 20220312. All methods were performed in accordance with the relevant guidelines and regulations.

Animal studies were conducted at Henan Key Laboratory for Pharmacology of Liver Disease.

## Data Availability

The datasets used and/or analysed during the current study available from the corresponding author on reasonable request.

## Abbreviations

EBRT: External beam radiotherapy
LD: Low dose
HD: High dose
VAR: Ventilate area ratio
QHS: Qualitative histological scoring
